# Tales From the Chalkface: Using Narratives to Explore Agency, Resilience, and Identity of Gay Teachers

**DOI:** 10.3389/fsoc.2020.00052

**Published:** 2020-07-24

**Authors:** Samuel Stones, Jonathan Glazzard

**Affiliations:** Carnegie School of Education, Leeds Beckett University, Leeds, United Kingdom

**Keywords:** queer, teachers, gay, stories, narrative

## Abstract

Existing literature is dominated by accounts which position gay teachers as victims. We were concerned that this only presented a partial insight into the experiences of gay teachers. This study researched the personal and professional experiences of four gay teachers in England. It builds on existing research by presenting positive narratives rather than positioning gay teachers as victims. We use the term “chalkface” to illustrate that all were practicing teachers. The purpose of the study was to explore their experiences as gay teachers throughout their careers. The study used the life history method to create narratives of each participant. Semi-structured interviews were used. The study found that the repeal of Section 28 in England in 2003 did not have an immediate effect on the identities, resilience, and agency of the participants. The 2010 Equality Act in England and changes to the school inspection framework had a greater influence in supporting their agency, resilience, and willingness to merge personal and professional identities. All but one participant managed to use their identities as gay teachers to advance inclusion and social justice through the curriculum. Although the narratives that we have presented do illuminate some negative experiences, the accounts are largely positive, in contrast with existing literature which positions gay teachers as victims.

## Introduction

This study explores the experiences of four gay educators who taught in schools during Section 28 and following its repeal. We were interested in exploring the ways in which Section 28 impacted on the agency, resilience and identities of these teachers during the time that the legislation was in force and following its repeal. Homosexuality was partially decriminalized in England and Wales in 1967. Despite this, the government of the United Kingdom introduced Section 28 in 1988 which prevented schools from promoting homosexuality or its acceptability as a “pretended family relationship” (Local Government Act, 1988). Research demonstrates that the legislation continued to impact and influence teachers' practice and identities for many years after its repeal in 2003 (Greenland and Nunney, 2008; Edwards et al., 2016). This study explores the participants' experiences of teaching during and after the repeal of Section 28. It explores the international literature on the experiences of teachers who are lesbian, gay, bisexual, trans, or queer (LGBTQ+). It explores theoretical perspectives on stress, resilience, identity, and agency. The complete narratives of the participants are presented because we wanted to privilege their stories. The narratives are subsequently analyzed using the theoretical frameworks that are outlined earlier in the paper.

### The Experiences of LGBTQ+ Teachers

Homosexuality was decriminalized in England and Wales in 1967. Prior to this, individuals engaging in homosexual acts faced a maximum sentence of life in prison. Despite decriminalization, official, and legal disapproval of homosexuality continued for many years with inequality remaining prevalent (Epstein, 2000; Nixon and Givens, 2007). Introduced by Margaret Thatcher's Conservative government in 1988, Section 28 (Local Government Act, 1988) signaled this disapproval by seeking to impose upon local authorities and their schools a prescribed view which sought to repress and restrict public debate of sexuality (Nixon and Givens, 2007). It has been argued that:

*Section 28 (part of the Local Government Act of 1988) was a notorious piece of legislation that sought to prevent local education authorities in the UK from ‘promoting homosexuality’. The effect of Section 28 was to create uncertainty and fear among teachers as to what was (and what was not) permitted in schools*. (Greenland and Nunney, 2008, p. 243)

Recent research demonstrates the powerful and long-lasting cultural effect of Section 28 (Edwards et al., 2016). It contributed to a climate of fear through the normalization of heterosexuality, thus resulting in marginalization, oppression, and regulation of those with deviant sexual identities (Neary, 2013). It has been emphasized that:

*Most research referring to [Section 28] has been highly critical, viewing it as symbolic discrimination that institutionalizes a hierarchical relationship between heterosexuality and homosexuality, and it is held up as a prime example of the exclusion of lesbians and gay men from full cultural citizenship*. (Burridge, 2004, p. 329)

Teachers held several misconceptions about Section 28, especially in relation to what was legal and what was not, and this uncertainty and confusion caused difficulties (Warwick et al., 2001). For example, teachers were often unable to draw distinctions between promoting homosexuality and simply providing students with advice (Greenland and Nunney, 2008). In addition, many teachers were unsure about the legality of discussing homosexuality, and this often led to an avoidance of the subject entirely (Buston and Hart, 2001). This meant that schools avoided discussion of LGBTQ+ topics and any related curricula (Epstein et al., 2003). Research also demonstrates that Section 28 supported the growth of homophobic bullying through creating school cultures which failed to challenge and address homophobia and homophobic harassment (Epstein, 2000; Warwick et al., 2001).

Section 28 prohibited schools from promoting homosexuality or its acceptability as a “pretended family relationship” (Local Government Act, 1988). This normalized heterosexual marriage (Nixon and Givens, 2007) and sustained cultures of heteronormativity in schools, despite the partial decriminalization of homosexuality over 20 years earlier. Thus, Section 28 reinforced the marginalization of people with LGBTQ+ identities. As demonstrated by Foucault (1978) and Ellis (2007), homo sexuality has been historically associated with disease and mental illness. Through condemning difference, Section 28 effectively positioned teachers with non-heterosexual identities as patients and sufferers (Ellis, 2007) whose divergence and difference left them feeling at risk and in need of help (Quinlivan, 2002).

Section 28 was repealed in England and Wales in 2003. Research demonstrates that the act continued to impact and influence teachers' practice for many years after its repeal (Greenland and Nunney, 2008; Edwards et al., 2016). Researchers have also argued that this repeal was a superficial change in legislation which only went a small way in challenging the deep heterosexist discourse and gross inequality already embedded in schools (Nixon and Givens, 2007). In part, this research study will explore the effect of Section 28 on teacher agency, resilience and identity and whether this has changed since its repeal in 2003.

Some research has demonstrated the harassment and discrimination of teachers with LGBTQ+ identities (Cooper, 2008; Neary, 2013). Dominant heteronormative discourses in schools often situate teachers with LGBTQ+ identities within exclusionary spaces (Gray et al., 2016). Research has linked these experiences of bullying, violence, invisibility, and alienation with elevated risks of mental ill health, self-harm, and suicidality (Mayock et al., 2009; Bryan and Maycock, 2017). Eliason (2010) conceptualizes the “suicide consensus” (p. 7) that has emerged from over 30 years of research. This research compared the experiences of individuals with LGBTQ+ identities with those of peers whose identities were normative (Bryan and Maycock, 2017). LGBTQ+ teachers are required to negotiate complex personal and professional boundaries (Vicars, 2006; Gray, 2013) and decide whether or not to be visible and open about their private truth (Grace and Benson, 2000). This isolation has deterred teachers from assuming positions as visible role models in schools (Russell, 2010; Gray et al., 2016). To conceal and reduce stigmatizing labels, individuals with LGBTQ+ identities will often pass off and cover up their sexuality in order to seek acceptance and equivalence. Through doing so, these teachers can conform to the heteronormative and heterosexist discourses that prevail in schools (Gray et al., 2016; Reimers, 2017).

According to Røthing (2008), teachers' experiences are influenced by “homotolerant” (p. 258) school cultures. Although heteronormativity might be less overt than it was previously (Berry, 2018), it still exists in subtle forms (Gray et al., 2016). These include bias and microaggressions (Francis and Reygan, 2016). Despite microaggressions originally emerging from race-based research (Lynn, 2002; Yosso, 2005), they have been explored in recent years in relation to sexual orientation and gender identity (Nadal et al., 2011; Francis and Reygan, 2016). Microaggressions therefore appear in a range of settings and contexts and can be understood as:

*…brief and commonplace daily verbal, behavioural, or environmental indignities, whether intentional or unintentional, that communicate hostile, derogatory, or negative slights and insults towards members of oppressed groups*. (Nadal, 2008, p. 23)

Research has demonstrated that the LGBTQ+ community, including staff and students in schools, is exposed to microagressions and subtle bias which perpetuate heterosexism and exclude those with LGBTQ+ identities (Walls, 2008; Nadal et al., 2011). Francis and Reygan's (2016) research has summarized the microaggressions facing those in the LGBTQ+ community. These include: heterosexist language; heteronormative and gender normative discourses; exoticising the identities of LGBTQ+ individuals; outright disapproval of those with LGBTQ+ identities; denying homophobia and pathologising those within the LGBTQ+ community. Minikel-Lacocque's (2013) research also characterizes the contested microaggressions which occur when aggressors deliberately and purposefully deny their actions.

Research demonstrates many of the factors contributing to the oppression of teachers with LGBTQ+ identities, including negative comments from students, peers, colleagues, lack of promotion, being forced to conceal their personal identities and heteronormative discourses in schools (Vicars, 2006; DePalma and Jennett, 2010; Piper and Sikes, 2010; Ferfolja and Hopkins, 2013; Gray, 2013; Gray et al., 2016). In addition to this research, teachers with LGBTQ+ identities have also been viewed with suspicion by parents and other adults (Rudoe, 2010) and recent safeguarding discourses has meant that a teacher's disclosure of their sexuality might be considered inappropriate (Gray et al., 2016).

Although there is a paucity of literature available (Ferfolja and Hopkins, 2013), research does demonstrate that the experiences of LGBTQ+ students have improved in very recent years with more students now self-identifying as LGBTQ+ to resist bigotry and discrimination (Berry, 2018). Despite this, research demonstrating the positive accounts of teachers in England remains sparse. Reflecting on the scarcity of this research, it is also important to consider the advances in international LGBTQ+ inclusion.

### The International Context

However, despite more liberal attitudes in some contexts, it has been argued that heterosexuality is embedded in the practices of institutions and the encounters of our everyday life (Epstein and Johnson, 1994). Although the rights of individuals with LGBTQ+ identities have been strengthened across Europe (Lundin, 2015), international research continues to demonstrate that heteronormative and heterosexist cultures are entrenched within schools (Kjaran and Kristinsdóttir, 2015). There is also evidence that these normative values are inculcated within schools in countries where homosexuality is legal, including Australia (Gray et al., 2016) and the United States (Lineback et al., 2016).

Even in countries known for their liberal attitude toward sexuality, such as Sweden, heteronormative attitudes continue to prevail within schools (Lundin, 2015). Furthermore, in countries where homosexuality is illegal or disapproved of, including some Asian and African countries, strict cultural values are used as a “yardstick” (Amoah and Gyasi, 2016, p. 1) to disregard the rights of those with LGBTQ+ identities (Po-Han, 2016).

Regardless of the legal status of homosexuality, religion, and culture shape public opinion on its acceptability (Adamczyk and Pitt, 2009). Research demonstrates that teachers with LGBTQ+ identities from across the globe continue to experience discrimination and marginalization (King et al., 2008; Hardie, 2012; Marris and Staton, 2016). Together, these factors restrict the willingness and ability of teachers to declare their sexuality in professional settings (Wright and Smith, 2015). This study will reflect on this and research the lived experiences of LGBTQ+ teachers in England.

The recognition of same-sex relationships in the United States has increased, though many individuals with LGBTQ+ identities continue to face discrimination (Lineback et al., 2016). Despite this societal tolerance, research has demonstrated that some schools in the US provide discriminatory environments for lesbian and gay individuals and that teaching is one of the most homophobic professions in parts of the US (DeLeon and Brunner, 2013; Lineback et al., 2016). According to DeLeon and Brunner (2013), attempts have even been made to exclude LGBTQ+ teachers from the profession to lessen the risks of sexual abuse, pedophilia, molestation, and the recruitment of children into queer lifestyles (Jackson, 2007; Mayo, 2008; Lineback et al., 2016). Individuals with discriminatory views have accused LGBTQ+ teachers in the US of attempting to influence students' identities and this illustrates the problematic and homophobic school cultures which some teachers in the US are exposed to Jackson (2007).

Recent research by Reimers (2017) draws on data from a Swedish teacher training programme and demonstrates how sexuality norms produce spaces of heteronormativity in which one body can be more vulnerable than another. According to Reimers (2017), Sweden provides an environment for “queers” (p. 92) which is better than in many other places, although identifying as LGBTQ+ is still seen as deviation. Therefore, it can be argued that whilst attitudes in Sweden are generally more liberal toward sexuality, heteronormative discourses still dominate their schools and the experiences of queer teachers within them. To address the vulnerabilities of those within the LGBTQ+ community, Reimers (2017) suggests investigating homonationalism. This involves intersecting LGBTQ+ rights with a country's democracy and ideology (Puar, 2013). Reimers (2017) therefore argues that homonationalism can be used as a vehicle to advance an inclusive agenda within Swedish schools by favorably associating the country's ideology with the rights of those within the LGBTQ+ community.

To be LGBTQ+ and to work as a teacher is to occupy a complex terrain and exist within a “space of exclusion” (Gray et al., 2016, p. 286). This draws on Vicars' (2006) concept of “problematic terrain” (p. 351). Although state schools in Australia protect those with LGBTQ+ identities, Gray et al. (2016) highlights that such protection is not offered by independent and religious schools and that teachers within these schools are obliged to uphold any religious ethos. Ferfolja's (2008) research demonstrates that within the Australian Catholic schooling system, contractual obligations and the threat of dismissal are used to silence those with LGBTQ+ identities.

Even in countries where homosexuality is legal, teachers with LGBTQ+ identities are still likely to be victims of institutional apathy and there is a disconnect between the recognition of LGBTQ+ rights by societies and the recognition of these rights within education (Gray et al., 2016). Research also demonstrates this disconnect in the healthcare sector with one in every eight of the 5,000 LGBTQ+ people surveyed reporting experiences of unequal treatment from healthcare staff (Bachmann and Gooch, 2017). There appears to be limited research presenting any correlation between the prejudice-based bullying in these sectors.

### Minority Stress

Meyer (2003) Minority Stress model has been used by mainstream psychologists to explain how minority status can impact on mental health outcomes for individuals who identify as part of a minority group. It is a particularly useful model for understanding the experiences of LGBTQ+ teachers because it identifies the various stressors to which they might be exposed to.

The model identifies different types of stress that minority individuals experience. Environmental circumstances such as poverty can produce general stressors (for example, financial stress). General stressors could also include the loss of a job, experiences of bereavement, or changes in family circumstances, such as divorce (Meyer, 2003). Distal stressors relate to the experience of stigma, prejudice, discrimination, victimization, and bullying by others based on an individual's sexual orientation or gender identity produces distal stressors (Meyer, 2003). These experiences can be shaped by structural forces (for example, racism, heteronormativity/heterosexism) which result in structural disadvantage for minority groups. Proximal stressors relate to an individual's perception or appraisal of situations. The expectation or anticipation that a person with a minority status may experience rejection, discrimination, victimization, or stigmatization based on one's previous experiences of this can result in self-vigilance and identity concealment (Meyer, 2003). LGBTQ+ teachers may anticipate negative reactions to their sexual orientation or gender identity from students, parents, or colleagues. To reduce the likelihood of negative experiences occurring, self-vigilance and concealment are employed but these tactics can result in fear of discovery, psychological distress, internalized shame, guilt, anxiety, and social isolation. Internalized negativity is where LGBTQ+ people internalize negative messages from others about their identities. It is a product of social prejudices. It can result in feelings of shame and self-disgust and can lead to adverse mental health outcomes (Herek et al., 1998; Herek, 2009). This can affect an individual's sense of self, resulting in detrimental impacts upon academic achievement, confidence, and social connectedness. Intersectional identities (for example, someone who is LGBTQ+ and has a disability) can result in multiple forms of discrimination.

Meyer (2003) identified social support systems as a vital factor in protecting minority groups from adverse mental health. Individuals may therefore choose to participate in sexual minority communities to enable them to enter into a non-stigmatizing environment (Cohen, 2004; Shechner et al., 2010). LGBTQ+ teachers may choose to join an LGBTQ+ network or they may form online social networks to gain support and positive affirmation. More recently, Meyer (2015) has argued that community resilience is an aspect of the minority stress model. LGBTQ+ people might access the queer community to benefit from community resilience. Meyer (2015) argues against a focus on resilience within individuals because it focuses attention on the individual's response to stress rather than the stressor itself, which is the social environment which the individual is exposed to. Research by Baams et al. (2015) found that feeling like a burden to significant others in their lives is a critical mechanism in explaining higher levels of depression and suicidal ideation among LGB youth. They found that although girls experience lower levels of stress in relation to coming out than boys, they felt more of a burden to family and friends and were therefore more likely to experience depression and suicidal ideation.

### Resilience

It is essential to consider the theme of resilience when exploring the capacities of LGBTQ+ teachers to navigate personal and professional transitions. Evidence suggests that resilience influences an individual's ability to adapt to transitions (Jindal-Snape, 2016). We conceptualize resilience as a characteristic that is not just individual but one that is relational. We draw on Greenfield's (2015) model of teacher resilience which examines the impact of relationships, institutional cultures, challenges, and the broader policy context on the resilience of teachers. This ecological framework of resilience is applied to the data to understand the factors which influence the resilience of the participants. This will address the final research question.

Traditional perspectives on resilience have conceptualized it as a fixed trait within individuals (Masten and Garmezy, 1985). However, more recent perspectives conceptualize resilience as a dynamic attribute which is influenced by social, cultural, and political contexts (Luthar, 2006; Roffey, 2017). Although some perspectives on resilience emphasize positive adaptation following adversity or trauma (Gayton and Lovell, 2012) and the capacity to grow in response to adversity (Stallman, 2011), these perspectives are not sufficient because they place emphasis on the individual to overcome adversity rather than exploring the systemic factors which directly influence a person's resilience (Meyer, 2015). Traditional perspectives emphasize resilience as the ability to rebound (McIntosh and Shaw, 2017), the ability to problem solve and to return to the previous state (McIntosh and Shaw, 2017; Sanderson and Brewer, 2017). This ability to push through regardless of circumstances is a dominant theme in the literature (Reyes et al., 2015) but these perspectives only offer a partial understanding of resilience because they do not acknowledge that resilience is relational and influenced by societal structures.

Literature has started to present models of resilience which identify the interdependency between the individual and broader contexts which intersect with their lives and the significance of these contexts in shaping resilience (Hartley, 2011). For example, Jameson (2014) provides one of the few accounts of resilience from a systemic perspective. In addition, Greenfield (2015) model of teacher resilience emphasizes the way in which teachers are positioned within social and broader contexts which impact on their individual resilience. Although this framework will be used as a conceptual lens within this study to analyse the factors which shape the resilience of the participants, the model fails to identify the specific contexts which shape teachers' lives. Examples of these include political factors which restrict or support teacher agency and religious discourses which may impact on teacher resilience for teachers who are working in schools with a strong religious affiliation or which serve religious communities. However, the model is useful in that it identifies the individual, relational, and contextual factors which can serve as both risk and protective factors in relation to a teacher's resilience. These include a sense of hope, purpose and self-efficacy (individual factors), relationships with family and friends (relational factors), relationships with leaders and other colleagues (contextual factors), the extent of the challenges which teachers face and the broader policy context in which teachers operate (Greenfield, 2015).

### Agency

In analyzing the lived experiences of the participants, this study will examine the extent to which their agency is restricted or otherwise by systemic factors and discourses which regulate their working lives. Evidence suggests that individuals with greater agency experience smoother transitions (Bandura, 2000, 2001). Although agency has been conceptualized as the ability to take initiative (Jindal-Snape, 2016) and make choices, it is important to emphasize that agency is context-specific (Jindal-Snape, 2016) and also influenced by one's self-efficacy (Bandura, 2000). The concept of teacher efficacy is particularly relevant to this study. Specifically, this study will draw on Pantić's (2015) model of teacher agency which identifies four factors that influence teacher agency. These include sense of purpose, competence, autonomy, and reflexivity to mediate or overcome barriers which restrict agency (Pantić, 2015). The model is useful because it positions teacher agency within the broader socio-cultural contexts in which teachers operate and therefore acknowledges the role of systemic factors in influencing teacher agency.

### Identity

Literature suggests that LGBTQ+ teachers navigate their personal and professional identities (Gray, 2013). Some participants may be in the process of coming to terms with their gender identities or sexualities and will make decisions about whether to separate or intertwine their personal and professional identities. Within the context of this study, identities will be viewed as multiple and exist within a state of flux rather than being conceptualized as unified, coherent, and static. LGBTQ+ teachers have personal and professional identities which can intertwine or collide. Research demonstrates that some choose to maintain a distinction between their personal and professional identities and others mesh them together by using their personal identities to advance LGBTQ+ inclusion in schools (Stones and Glazzard, 2019). In addition, some teachers may adopt Goffman's (1963) techniques of “passing” (p. 73) or “covering” (p. 102) to conceal their non-normative identities.

Seminal work on teacher identity has illustrated how the occupational and personal selves become integrated to produce a coherent self (Nias, 1989) whereas other work has highlighted the tensions that exist between substantial and situational selves (Sikes et al., 1985; Woods and Jeffrey, 2002). However, more recent work suggests that teacher identity is not a stable entity but continually reconstructed as a product of conflicting discourses and practices (Sikes et al., 1985; Day et al., 2006). It is always deferred and in the process of becoming: “never really, never yet, never absolutely there” (MacLure, 2003, p. 131). Thus, identity formation is a continual process of negotiation and “a potential site of agency” (Clarke, 2009, p. 187).

### Research Gaps

Existing research has considered the experiences of LGBTQ+ teachers. A synthesis of this literature highlights recurrent themes including marginalization, bullying, harassment, discrimination, and isolation. There is research from countries where homosexuality is illegal or disapproved of, including some African and Asian countries (Amoah and Gyasi, 2016; Po-Han, 2016), as well as in countries where homosexuality is legalized, such as Australia (Gray et al., 2016), the United States (Lineback et al., 2016) and throughout parts of Europe (Lundin, 2015).

Despite this international literature, there is a paucity of research capturing the experiences of LGBTQ+ teachers and who currently teach or have taught in schools in England. Much of the existing research positions those within the LGBTQ+ community as victims (Gray et al., 2016) who are exposed to suffering and violence (Devís-Devís et al., 2017) and there appears to be limited research presenting positive accounts despite the field of positive psychology which has grown significantly in recent years (Lytle et al., 2014; Pawelski, 2016).

### Research Aims and Questions

The broad aim of the research was to explore the experiences of gay teachers who taught during and after Section 28 in England. We wanted to explore the ways in which the legislation impacted on them and whether their experiences changed following the repeal of the legislation in 2003.

This research study addressed the following research questions:

- What have been (and currently are) the experiences of LGBTQ+ teachers?
- What factors affect their resilience?
- How do they negotiate their personal and professional identities?

Much of the existing literature positions queer teachers as victims who lack agency and are forced to conceal their identities (King et al., 2008; Hardie, 2012; Marris and Staton, 2016) or maintain a separation between their personal and professional identities (Wright and Smith, 2015). This study sought to offer counter-narratives to the victimized narratives which are dominant in the existing literature.

## Methods

This research study explored the lived experiences of four gay teachers. We sought to capture the unique nature of people's experiences (Goodson, 1992; Goodson and Sikes, 2001) in a form that was both engaging and compelling. Within a narrative methodology, we used the life history method to illuminate the unique and rich experiences of an individual's life (Webster and Mertova, 2007; Riessman, 2008). This method places informants' stories within the broader context of public issues and in doing so highlights the social and cultural discourses which intersect with the lives of individuals. Each teacher participated in a semi-structured interview.

### Participants

Participants were recruited to the study using our personal social media platforms. We were interested in representing the stories of queer teachers and we put out an open call to invite participation. The criteria for inclusion were that the participants needed to identify as lesbian, gay, bisexual or trans, that they must be practicing teachers and that they must have taught during Section 28. Unfortunately, we did not secure participation from teachers who identified as lesbian or bisexual or trans and we recognize that this is a methodological weakness of the study. We have reflected on our own positionality within the research (Berger, 2013). It is possible that our own status as two gay male researchers impacted on the diversity of the sample. None of the participants were known to us. The breakdown of participants is included in Table 1.

**Table 1:** Participants.

	**Sexuality**	**Gender**	**Teaching sector**	**Type of school**	**Role**	**Years of experience**
Tom	Gay	Male	Primary/higher education	State	Teacher/lecturer	25
Jack	Gay	Male	Primary	State	Head teacher/principal	22
William	Gay	Male	Secondary	Independent	Senior leader	23
Oliver	Gay	Male	Secondary	State	Senior leader	20

### Procedures

We used semi-structured interviews in which we simply invited each participant to tell us about their experiences of being a queer teacher. Interviews were conducted via video conferencing software and audio recorded. We did not use a schedule but decided to follow the lead of the participants' (Alasuutari et al., 2008). Data were captured using digital recordings and transcribed to create life narratives for analysis.

The study gained ethical clearance from the university ethical approvals committee. Informed consent was gained prior to collecting any data and participants were assured of their rights to confidentiality and anonymity.

As Laurel Richardson has pointed out, writing about and re-presenting lives carries a “moral responsibility” (Richardson, 1990, p. 131) and consequently “it is not to be embarked on lightly” (Goodson and Sikes, 2001, p. 99). We were committed to using our “narrative privilege” (Adams, 2008) wisely by jointly interpreting data with participants and using member checks after the accounts were constructed.

### Data Analysis

Thematic analysis was used using an established framework (Braun and Clarke, 2006) to identify key themes arising from across the four narratives. Firstly, each narrative was analyzed individually to identify emergent themes. A cross-sectional analysis was then carried out to identify common themes from across the narratives. The outcomes of the cross-sectional analysis are presented in Table 2.

**Table 2:** Cross-sectional analysis.

**Tom**	**Jack**	**William**	**Oliver**	**Final themes**
Teacher agency Violence Teacher identity Being outed Resilience Stress	Teacher agency Power Teacher identity Geographic displacement Resilience Stress	Teacher agency Teacher identity Resilience Deep inclusion Stress	Teacher agency Religion Teacher identity Resilience Stress	Teacher agency Teacher identity Resilience Stress

A table of themes arising from the analysis is presented in Table 3.

**Table 3:** Aligning themes with the data.

	**Examples from the data**
**Identity**	*When I started teaching in 1996 under Section 28 the culture was very different. I felt it might be an issue for staff and children, so I didn't say anything. My first school was in Leeds. I lived in Manchester, so it was easy to keep my personal and professional lives separate. I made a conscious decision to look for jobs on the other side of the Pennines (Jack). When I first started teaching in the 1990s the law hadn't changed. I didn't lie but I only came out to some colleagues. I got the sense that if I pushed too far I would be pulled in for a conversation. I could have got the sack (William). Tom knew he could separate his personal and professional life and that he would not need to discuss his sexuality with colleagues and students. Hiding the truth gave Tom a safety net. He felt a sense of protection (Tom). My sexual orientation does not come into my teaching. Our focus is to educate and teach. My ethnicity has shaped my career more (Oliver)*.
**Agency**	*The repeal of Section 28 has changed things, but it has been a delayed reaction. Much of the change didn't happen in 2003 and it took time, but the repeal resulted in changes to equality legislation in 2010 and changes to the Ofsted framework after that (Jack). I don't feel able to be open. My Vice Principal is a lesbian and she isn't out either. I would not be comfortable being out in the role I'm doing with the community that I serve. It is a predominantly Muslim community which makes it more difficult to prioritize a culture of acceptance … I know students suspect and have said things behind my back and some of the male staff of a particular ethnic faith have some issues about sexual orientation (Oliver). When I got my job in 2011, a small group of evangelical Christians said to the Head, we think you have just appointed a gay and we are not happy about it. The Head was horrified. I decided I wasn't going to edit myself out, partly because heterosexual staff don't edit their lives, but also partly to watch the fear behind their eyes (William). I was open about my sexuality from day 1 in my current school. I'm the Head so there is no one higher than me (Jack). I have freedom in the university to be open with students and colleagues about my sexual orientation. As a teacher educator I feel able to teach my students about issues pertaining to sexuality or gender identity in schools because this is a requirement of the Equality Act and school inspection frameworks (Tom)*.
**Resilience**	*I told him about the law and said to him, if you are not happy you can take your child elsewhere (Jack). If I get backlash from parents, I just say, it's the law (William). If I can wrap LGBTQ+ issues up with the Equality Act, I will. I find it easier to talk about LGBTQ+ alongside other protected characteristics. I won't say things that will identify me. Comments were made about me by a colleague in the junior team which were hurtful. I am not out to all staff. I was told by my [line manager] not to go flaunting it around (Oliver)*.
**Stress**	*In 2005 it was the early stages of my headship. The local authority had shortlisted the applications and I went to the teachers' center to collect them. One of the applications had a big star in the corner. I questioned this and was told that the feedback from the shortlisting panel was that this candidate was obviously gay. The local authority officers were endorsing homophobia after the repeal of Section 28. I thought, well I'm gay, I'd better be careful (Jack). I came out to colleagues but not explicitly to pupils … I got the sense that if I pushed too far I would be pulled in for a conversation (William)*.

## Results

The following narratives were produced using the interview transcripts. The narratives have been developed from the transcriptions and do not include all aspects of the transcriptions.

### Tom

September 1990 had arrived. It had been mid-day on the first day of a new school year and the high-pitched chime of the lunchtime bell had reverberated through the corridors. Tom was in his third year of secondary school and his routines had been well-rehearsed. He often left school to buy his lunch and flee the terror of the school canteen. Although he hadn't realized, his efforts to escape one horror had been exposing him to an even greater evil. Tom had always known that one day he would feel like he was trapped inside a burning building. In his nightmares, he saw a building with no exit and no escape route. Tom became visibly upset when he recalled the incident described in this vignette.

The weather was bitterly cold and my hands and feet were freezing. The sky was dull and the air was thick. The pounding rain was not enough to block out the smell of noxious smoke oozing from the tall chimneys of the long rows of terraced houses. It was overbearing. We lived in a former mining community—this was a place where men were meant to be men! Oliver and I were walking back to school. Going into town for lunch was a way of escaping the pain and misery that we would have endured had we eaten in the school canteen. I had lost count of the number of times I had been called “a fucking gay faggot.”

Suddenly, Simon ran up behind us. I didn't see him coming and I certainly didn't expect it. I thought that the bullies congregated and ate together at school. He was short and spotty but he held a reputation for being tough. He began punching me in the head and I crashed to the ground. My head hit the pavement and I blacked out. I gained consciousness but I could not see Oliver. Perhaps he had gone to get help. Blood was streaming down my face like a gushing waterfall. I could feel my eyes swelling as Simon continued to kick me repeatedly in the stomach.

Simon started stamping on my head. “Die you fucking queer, you deserve to get AIDS.” The pain was unbearable, and I used my hands to protect my head. My head began to throb as though I had been hit by a car. I pleaded with him to stop and let me go but he was wound up and roaring at me like a caged tiger. I curled into a tight ball trying to protect my body. I could hear the traffic screeching past, but no-one stopped. It lasted all of a few seconds, but it felt like hours. He crouched down and screamed right into my face. “Queer! Arse-fucker, cock sucker, stay away from me.” The abuse continued. I felt trapped and Oliver had not returned. He had run off when Simon began punching me.

I felt dirty and ashamed. At one point, I wanted him to kill me. After all, I knew that I could not tell my parents what had happened because I wasn't out to them. My father would have been disgusted. He had made his feelings clear. I knew my mother would be more understanding because she worked in a gay nightclub, but I could not be sure she would accept me being gay. I knew I couldn't report it to the police, because they hated people like me.

All of a sudden a woman raced over the road and yelled at Simon. He stopped and cowardly ran away toward the school. She checked I was conscious, and I got up and made my way back to school, terrified that he would be waiting round the next corner to finish what he had started.

The thought of meeting him again in school and of what he might do to me made me feel sick to the pit of my stomach. I decided to tell my form tutor, Mr. Orange, what had happened. Mr. Orange was a decent man and a good English teacher. He once jokingly chastised me for writing “chocolate” on the front of my English book in front of his surname. He could have ripped me to pieces but he didn't. I knew he liked me. I edited the bad language out of my account but told him the rest of what had happened. He knew I wasn't lying because my face was still covered in dried blood and dirt from the pavement. He listened patiently and his reply shocked me to the core. “Tom, there is nothing we can do because this took place outside of school. Just watch where you go and stay away from him.” I had never felt safe in school, and I now knew that this would never change.

After years of suffering as a student, Tom wanted to make a difference. He wanted to be able to empower young children and make sure that they did not suffer throughout their own schooling, like he had. Tom knew he needed to train and become a teacher. After a 4 year course Tom was excited at the prospect of having his own class. It was 1998 and Tom had secured his first interview for a teaching post. He had completed his teaching practice placements in large schools located in sprawling council estates. However, Tom's interview was at a village primary located in a beautiful rural area. Immaculately maintained lawns fronted large detached houses, with luxury cars sat prominently on their drives. He was not used to places like this and already felt out of his depth. His sexuality strengthened these anxieties and he feared that his identity would impede his success. Tom was walking down a dark alley and knew nothing about what was waiting for him ahead. In the following vignette, he recounts his vivid memories of the recruitment and selection experience.

It was a hot and sunny day in May. I had to catch a train and a bus to get to the school as I didn't drive. The interview was one of those grueling scenarios. It involved meeting the staff, having lunch and talking to other candidates while sitting around all day waiting to be interviewed. The lunch was a disaster because it was dairy and meat. I am vegan but was too scared to say anything in case it made me stand out or look odd. I couldn't afford this at interview and I ate the meal. Sat opposite a panel of 12 interviewers, I then began to answer questions as they were fired at me one-by-one.

“Why have you applied to be a Reception teacher?” The question took me by surprise. There was an emphasis on why I wanted to teach young children and not why I wanted to teach. The Chair of Governors was a fat, obnoxious man with dark rimmed spectacles and a receding hair line. “I want to teach kids to read and write and give them a really good foundation.” When chatting to other candidates, I realized that they hadn't been asked this question. That realization made me feel uneasy.

After the interview there was a torturously long wait. Suddenly, I was startled out of my thoughts. “Tom, the Head is ready to see you.” Walking toward his office, thoughts raced through my mind. “Your application for this post has been unsuccessful.” I didn't get the job. “We don't think you will fit into a school like this. We're in a very middle-class area and the parents here are really fussy.” My mind was flooded with emotions. Was it because they knew I was gay? Did they think I was too camp? Did they dislike the way I walked or talked? Why had the Chair of Governors asked me that question? Did they think I was a pedophile? I was the best student on my 4-year teacher training degree. I achieved distinctions in all of my teaching practices and I won the course prize for academic achievement. Why would I not fit in? I had never experienced rejection like this before.

Then came an about-turn. Dianne contacted me 3 days later. She had been one of the teachers who had interviewed me. “Tom, that job was yours. You scored the highest points in the interview.” The Chair had blocked my appointment. “We cannot have a homosexual teaching in this school. What will the parents think?” Dianne thought that I should know.

I was absolutely furious. I was not taking this news lying down. I wasn't going to let someone who knew nothing about education ruin my career. A career that I deserved! I contacted the local authority and asked for the interview records to be recalled and scrutinized. I had been discriminated against and I had to make a stand. I felt it was my duty to all the other teachers like me. Teachers who wanted to commit their working lives to education. I eventually received an embarrassed apology from the local authority and was offered the job. I didn't want to work there but I needed the job so reluctantly I accepted. There must have been some serious hand slapping that week although to my dismay no one lost their job.

Despite the challenges he faced in securing a teaching post, Tom felt reassured. He knew he could separate his personal and professional life and that he would not need to discuss his sexuality with colleagues and students. Hiding the truth gave Tom a safety net. He felt a sense of protection. He remained in that school for a decade and only disclosed his sexuality to colleagues he felt he could trust. He then moved into an academic career in higher education where he was able to openly disclose his sexuality and merge his personal and professional identities.

### Jack

It was 1996 and Jack was looking to secure his first teaching post. He had lived in Manchester for most of his life though he knew he could never work there. He was only applying for posts in Leeds. It was an easy decision for Jack to make. He felt he needed a role on the other side of the Pennines so that he could separate his personal life from his job. He felt unable to bring the two together. When he had been looking for jobs, he never considered any in Manchester. After several years of working as a primary teacher, he moved schools and became a senior leader. He did not come out in his new school. The thought of doing so made his heart race. He feared that members of staff and governors might have an issue with it. He couldn't afford that. In 1996, the culture was very different under Section 28. He felt that his sexuality might be an issue for staff and children. He never told them. As a Deputy Headteacher and Headteacher, Jack had responsibility for staff recruitment. In this vignette, he describes an incident he will never forget.

Early in my career as a Head we used a local authority pool system for teachers to apply for jobs. Teachers applied to a pool and could be recruited to work in any school in the authority. The local authority did the shortlisting and then the Heads looked at the application forms of those who had been shortlisted and offered interviews in their schools. I remember in 2005 going down to the teachers' center to look at a batch of shortlisted application forms. We needed a newly qualified teacher and I was desperate to appoint someone to the role. I pulled out one application form and I was puzzled why someone had drawn a big star and a circle on it. I questioned what this meant. “What do these annotations mean?” The local authority officer replied straight away without hesitation. “The candidate was worthy of being interviewed but the shortlisting panel felt it necessary to draw attention to the fact that the candidate was obviously gay.”

In a heartbeat, memories and feelings came flooding back to me. It was 2005 and the local authority officers were endorsing homophobia. Section 28 has been repealed but its legacy still cast a shadow. I was appalled and scared. I am gay. I need to be careful. Section 28 was repealed in 2003 and I saw very little in terms of change. There was very little change at that time anyway, but I knew that Ofsted would not have prioritized LGBTQ+ inclusion without Section 28 being repealed. Some changes did happen, though these took many years. Equality legislation and the revisions to the Ofsted framework provided some momentum. When Section 28 was repealed, people were still scared. Schools could now talk about gay people, but many were too frightened to do so for several years.

Jack has now led his current school as Head for 10 years. He decided to come out to staff and students immediately after his appointment. At that time, he had never anticipated being able to drive an agenda to promote LGBTQ+ inclusion. He knows that attitudes have changed significantly in recent years although the fear of parental backlash has stayed with him for 10 years. He now seeks protection through his role as Head. He knows that there is no one higher than him to halt the work he is doing to promote LGBTQ+ inclusion. Jack describes his work in this vignette.

We started this work 5 or 6 years ago. Back then, things were different. “You're gay.” “That's so gay.” The word “gay” was used by students as the insult of choice. It meant rubbish, bad, broken, and stupid. Boys who were not interested in football were often subjected to homophobic bullying. The culture was toxic. The bullying was endemic. We had to act. I felt the weight of responsibility. I had to lead this change and I was now responsible for its success. We worked with an LGBTQ+ charity to develop staff confidence. Our work raised the profile of LGBTQ+ inclusion and some bullying stopped although I continued to drive change with commitment and momentum. Kids stopped using the word “gay” because they knew that there would be consequences. We tried to normalize LGBTQ+ identities as much as possible. The governors were on board and they believed in our final destination. They shared my vision and they had responded well to LGBTQ+ training. Initially, we didn't highlight this work to parents. It was on our website, though I was too scared to make a thing of it.

We are now building a snowball. Each generation is more accepting than the previous generation. LGBTQ+ visibility in society continues to improve and this drives further advancement. People are beginning to understand how LGBTQ+ identities can exist within family structures. Parents are less likely to complain. My school is in an area of social deprivation. Some parents come from black African heritage and wanted me to explain the work we were doing. Some of my parents are racist. They know it is not acceptable to be racist on school grounds and it is exactly the same for LGBTQ+. I am not going to stop advancing inclusion simply because they do not like it.

Jack now collaborates with other schools who are developing their LGBTQ+ inclusion policies. Although he has developed and advanced LGBTQ+ inclusion in his own setting, he knows that the national picture remains variable and inconsistent. His work with other schools continues to reveal staff resistance and that many schools are facing challenges. In this final vignette, he describes the fear factor.

I work in other schools. There is still some apathy from staff. “We don't have a problem here.” Getting some staff to see the value of this work can be a challenge sometimes. It can be difficult to get them to realize that it is not just about doing a one-off lesson. It is about the ethos, culture and the curriculum of the school. It is not about ticking a box. I ask big questions to support their thinking and reflection. “What challenges do you face in relation to LGBTQ+ inclusion.” “The parents.” It is in the Ofsted framework yet there is still a fear factor.

In 2018 we ran a rainbow day. We showed the children videos of Pride and we hosted a whole school Pride parade. We had posters and banners. I was worried at the time that it would end up in the Daily Mail. A couple of parents came into school to complain about our “themed days.” One of them said he had an “issue” with it. I told him about the law and Ofsted. “If you are not happy then you can take your child elsewhere.”

### William

It was late 1990s and William was teaching in the independent sector. It was a boarding school and he lived in the boys' boarding house. He didn't lie about his sexuality if people asked him although he did try to keep it low key. He came out to some colleagues and he knew he could never tell pupils. His school was supportive though he sensed that if he pushed it too far the school would pull him in for a conversation. Everything was always at stake. He constantly worried that he could be dismissed for being gay. The secrecy was always there. In this vignette, William describes his move into middle leadership.

I moved into a middle leadership role after 9 years. It was another independent school and the year was 2010. I just thought to myself “this isn”t good enough; I'm not going to edit myself'. When I got the job a small group of evangelical Christians had spoken to the Head about my appointment. “We think you have just appointed a gay and we are not happy about it.” The Head was horrified and ordered them out of the office. When I arrived, I treated them kindly. I wanted to watch the fear behind their eyes. I am from a faith background and my husband is from India so I want people to understand that you can have a religion and also be gay.

I am completely open about my sexual orientation and this school has been wonderful. My husband and I got married in the school. I sometimes experience a little bit of homophobia. It is the casual language that pupils use. “That's gay.” “This is gay.” I pick up on it calmly. “As a gay man I find that offensive and I'd rather you didn't say it.” One boy spent a whole week trying to apologize to me. I have experienced a bit of resistance from staff. Some have implied that I have a personal agenda. It doesn't bother me because I have support from the Head and Deputy.

I talk about my personal life with my husband in school. If it is acceptable for a heterosexual colleague to bring their personal lives in to school, it is also acceptable for me to do the same. Our personal and professional lives overlap because this is a boarding school. We spend a lot of time with our pupils and they like to get to know us. Some colleagues have told me to keep my private life separate from my work life. I always give the same response. “You don't, why should I?” Sometimes I get excluded from heterosexual conversations, so I tell people straight that they are excluding me. I often tell people that it is okay for them to ask me questions about my life. Sometimes they treat my life as a taboo subject, which it isn't.

William now leads LGBTQ+ inclusion in his school. He's led surveys with parents, pupils and staff and he implemented an LGB policy and a separate transgender policy. He believes that the repeal of Section 28 made no difference to LGBTQ+ inclusion. In this vignette, he explains how the Equality Act (2010) gave him opportunities to advance inclusion.

The Equality Act in 2010 reversed the damage of Section 28, not its repeal. As a result of the Equality Act I have done a lot of work on LGBT inclusion in the school. We have embedded LGBT identities into the curriculum to increase visibility. I have invited LGBT role models into the school. I am a Stonewall school champion now and I support other schools with LGBT inclusion. Sometimes I get backlash from parents. I always refer to equality legislation during my conversations with parents. We introduced a gender-neutral dress code and one father complained. “All the boys will be wandering around in fishnet tights.” “That says more about how you feel about women than anything else.” I have organized a knowledge-exchange conference for schools to come together and share ideas and I invited LGBTQ+ students.

We don't do things that are over the top, such as launching a drag show! We have not created an LGBT group because this just becomes a gay ghetto and excludes those who are not ready to come out. Instead, we have set up an equality group which includes LGBTQ+ pupils. We write an annual report to governors and audit school policies to make sure they are LGBTQ+ inclusive. We have trained all staff in how to respond to LGBTQ+ bullying and we have included books in the library that are written by LGBTQ+ authors, address LGBTQ+ experiences and LGBTQ+ identities. We don't do drop-down days as we embed it through the whole school. We have changed application forms to make them gender neutral and we create opportunities for LGBTQ+ role models to visit the school. I want it to be boring, routine, and humdrum so that it is ordinary and just run of the mill. You need someone in the school to drive it. It doesn't have to be an LGBTQ+ person, but it kind of does! You need someone to lead it who understands the issues. It has to be part of their lived experiences. You can do it hypothetically but there is an emptiness to it. It would be a bit like having men trying to organize a women's rights movement.

William knows he's lucky to work in a school that proactively promotes LGBTQ+ inclusion. In this final vignette, he describes the current challenges that many schools still face.

The biggest issue is lack of time and finding the space to do this essential work. I have spoken in Muslim schools, schools in areas of social deprivation or schools where there are gypsy pupils. In those schools I have faced higher levels of resistance and aggression. I know a colleague who works in a Catholic school and they [senior leadership team] have told her not to speak about her sexuality. They have forced her into the closet. She has experienced homophobic abuse from pupils because they [senior leaders] are covertly condoning it. Some people think that addressing issues of sexuality is teaching pupils about sex. We are not sitting kids down and telling them to have gay sex. We are teaching them about identity.

### Oliver

Oliver started teaching in the late 1990s. His identity as a gay man doesn't come into his work and he has never been open about his sexual orientation. In this vignette, Oliver vividly recounts some painful memories.

When I was appointed as a Deputy, I filled in the equal opportunities form and identified as gay. I was then asked directly in the interview if I was gay. Another colleague told me that I should have walked out at that point. In 2015 I was appointed in an interim Head role and both Executive Head Teachers were black. “I noticed on the form that you are gay, don't go flaunting it around.” I didn't last long in that role because her values clashed with my own.

In my current school I am not able to be open. My Vice Principal is also a lesbian and she is not out. We are not a faith school, but the pupils are predominantly Muslim, and there are cultural traditions in the community. It is an area of high crime so we must prioritize other things such as behavior and safety. It is a small school so the capacity of the staff to do things is seriously stretched.

My sexual orientation does not come into my teaching. My focus is to educate and teach! As a Head I would not be comfortable being out, due to the role I'm doing and the community that we serve. One pupil came into our school and he was openly gay. He was teased and taunted. There was a lack of respect toward him. We have a high percentage of Muslim students. It is fine to be gay as long as you are not practicing the faith. I have never been out with my students. I'm happy to do LGBTQ+ history and LGBT Pride but that is about it. I am not out to all staff. There are some staff who I would not trust.

I experienced homophobia in my first middle leadership role in an independent school. I once went for an interview and there was one Asian candidate, one black African candidate and one white British candidate. It made me think about whether I was being judged for the role on my merits or whether they were just trying to tick boxes. I wonder how many Heads are LGBTQ+ because that is never talked about. However, we need to represent diversity in school leadership teams.

I know that my students suspect and have said things behind my back. Some of the staff in my school see LGBT as a taboo topic and are not happy to teach it. Some of the male Muslim staff have some issues with specific protected characteristics which they are not prepared to promote. I am not prepared to lead on LGBTQ+ inclusion. The Vice Principal leads on it. Sometimes it is done through a token gesture by addressing LGBT history month or doing an assembly on it. The curriculum has to serve the context of the school so LGBT inclusion is not a priority for me. The focus is on keeping the children safe!

## Discussion

The themes of identity, agency, and resilience were identified as common themes across the four narratives. Four decades ago, Goodson (1980) stated that “in understanding something so intensely personal as teaching, it is critical we know about the person the teacher is” (p. 69). It has been argued that ‘professional work cannot and should not be divorced from the lives of professionals’ (Goodson and Sikes, 2001, p. 71).

### Teacher Identity

It has been argued that teacher identity is neither static nor coherent but that it is fragmented and always in a state of flux (Smith, 2007). Thus, teacher identity is not a stable entity. Instead, it is continually reconstructed as a product of conflicting practices and discourses (Sikes et al., 1985; Day et al., 2006). It is “always deferred and in the process of becoming—never really, never yet, never absolutely there” (MacLure, 2003, p. 131).

Tom, Jack, and William's had actively chosen to intertwine their personal and professional identities and had decided to use their personal identities to advance LGBTQ+ inclusion within their schools. However, Jack, William, and Tom all separated their personal and professional identities when they started teaching in the 1990s. In the initial stages of their careers, they felt restrained by the force of the heterosexual matrix (Butler, 1990) which was upheld by Section 28. They experienced a culture of compulsory heterosexuality (Rich, 1980) and their stigmatized identities were displaced (Vicars, 2006). They negotiated their sexualities in school in various different ways. These included being selectively out to colleagues but not students (William) or covering up (Goffman, 1963) their sexuality and personal identities (Tom and Jack).

They made a deliberate decision to intertwine their personal and professional identities later in their careers, following changes to legislative and other regulatory frameworks which provided them with protection and permission to advance LGBTQ+ inclusion within their educational contexts. Literature has highlighted how teacher agency and identity are inter-related (Barcelos, 2015). Tom, William, and Jack were able to allow their personal and professional identities to overlap. They used their identities to support their efforts to promote LGBTQ+ inclusion. In contrast, Oliver maintained a division between his personal and professional identities which restricted his agency.

Seminal work on teacher identity has illustrated how the professional and personal selves become integrated to produce a coherent self (Nias, 1989) whereas other work has highlighted the tensions that exist between substantial and situational selves (Sikes et al., 1985; Woods and Jeffrey, 2002). Although Tom, Jack, and William had integrated their personal identities to produce a coherent teacher identity, this was not the case for Oliver who felt compelled to hide his personal identity due to strong religious community that his school served. Teaching assigns on educators a social identity which links teacher effectiveness with the ability to maintain a commitment to improving educational outcomes (Jeffrey and Troman, 2012). For Oliver, this social identity was more significant to him than his sexuality. Oliver believed that his primary role as a leader was to focus on raising educational standards rather than focusing on his own sexuality and advancing LGBTQ+ inclusion. Clarke (2009) argues that teachers have an ethical obligation to reflect on their identities and to engage in identity work by “claiming” their identity. However, this is not always possible, and this was evident with Oliver who, despite legislation which offered him protection, felt it necessary to maintain a clear separation between the different aspects of his identity, resulting in a fragmented and non-authentic identity during his work as a teacher.

Webb and Vulliamy (2006) have demonstrated how teachers are able to subvert, reject, and recast the dominant political versions of what it means to be a teacher, thus enabling them to assert their own professional values on their identity. Clarke (2009) argues that it is possible for teachers to author their own identities and William, Jack, and Tom each managed to do this successfully, despite having their identities constrained in the early stages of their teaching careers. The equality legislation and inspection framework supported their confidence in disclosing their personal identities in school, advancing LGBTQ+ inclusion, and negotiating parental resistance. It therefore seems that identity formation is a continual process of negotiation and “a potential site of agency” (Clarke, 2009, p. 187) but the extent to which teachers are assigned agency is influenced by the contexts in which teachers work.

### Agency

Pantic's model of teacher agency (Pantić, 2015) includes four factors that influence agency. Firstly, the teacher's sense of purpose is critical to their agency. Tom, William, and Jack all demonstrated a clear sense of purpose which was centered on promoting equality and social justice. Secondly, teacher competence facilitates or restricts agency. All participants had achieved senior or middle leadership positions in education. Although Oliver's agency was restricted by religious discourses, William, Jack, and Tom were assigned agency because they were competent teachers who were capable of developing whole institutional approaches to LGBTQ+ inclusion. Thirdly, autonomy was identified as a critical aspect of teacher agency. Tom, William, and Jack were given considerable autonomy to develop their work on LGBTQ+ inclusion. They were trusted by their line managers and the degree of autonomy which they were assigned allowed them to be agentic. This was not the case for Oliver. Finally, the model includes reflexivity which denotes the ability of the teacher to mediate or overcome barriers that obstruct their sense of purpose. This emerged strongly in William's narrative when he encountered staff and parental resistance to his work. His ability to resist these obstacles meant that his agency was not restricted. Jack also skilfully challenged parental resistance to his agency so that his sense of purpose was not detrimentally affected.

### Resilience

Greenfield (2015) model of teacher resilience demonstrates how resilient teachers have a sense of hope, purpose, and belief in themselves as teachers (self-efficacy). These core beliefs are individual characteristics which play a critical role in resilience. The model demonstrates how resilient teachers form meaningful relationships with others within their setting and undertake actions to effect change and mediate the challenges they face. The model demonstrates how wider systemic factors also influence resilience.

Tom, Jack and William demonstrated a deep commitment to equality and social justice. This motivated them to advance LGBTQ+ inclusion within their contexts. All four participants were highly successful educators and in relatively powerful positions. Their teacher-efficacy was high, and this supported them to be resilient to the challenges they faced. Relationships with colleagues were critical to their resilience and the work they undertook (actions) within their schools was critical to sustaining their motivation. Jack and William both faced challenges from parents and William also faced challenges from other staff with strong religious views, but the protection they were provided by the Equality Act (2010) and by the Office for Standards in Education Ofsted (2018) Framework enabled them to be resilient to these challenges. In contrast, Oliver's resilience was detrimentally affected by the religious context of the school in which he worked.

### Minority Stress

All participants had experienced a degree of minority stress at specific points in their careers. In some cases, distal stressors were caused by the actual experience of prejudice or discrimination. Tom was bullied for being gay and experienced direct discrimination during his interviews for teaching posts. Oliver was directly asked about his sexual orientation during a teaching interview and instructed to repress it. William experienced discrimination from other staff upon his appointment and Jack had experienced prejudice from parents. All participants had experienced the pressure to negotiate their sexuality during their early teaching careers and anticipated negative reactions to disclosures of their personal identity (proximal stressors).

All participants drew on the support from family, friends, or other networks to mitigate the effects of stress. The Equality Act (2010) and the Ofsted inspection framework resulted in Tom, William, and Jack feeling confident in merging their personal identities with their teacher identities. All participants had secured leadership positions in various sectors of education, and this gave them high levels of teacher efficacy which mitigated the effects of minority stress. The protection offered by the legislative context increased their resilience and reduced the effects of minority stress, with the exception of Oliver who experienced minority stress as a result of the religious context in which he worked. The positive institutional ethos and culture which Tom, Jack, and William experienced mitigated the effects of minority stress.

The findings suggest that it may be possible to adapt Meyer (2003) model of minority stress by including a wider range of coping strategies which mitigate the effects of minority stress. Meyer (2003) included social support as a coping mechanism but the data suggest that legislative and other policy frameworks (for example, inspection frameworks) can increase resilience and mitigate stress. The data also suggest that high levels of self-efficacy and positive institutional cultures can also mitigate stress.

## Conclusion

The narratives demonstrate that Section 28 had a detrimental impact on the teacher agency of all participants. Consequently, in the early stages of their teaching careers, the participants were forced to conceal or negotiate their sexualities in school. The repeal of Section 28 did not immediately result in greater teacher agency, nor did it allow them to intertwine their personal and professional identities to produce a coherent teacher identity. Greater agency was assigned following the introduction of equality legislation and regulatory frameworks for school inspections. These developments supported Tom, Jack, and William to author their own identities as teachers by merging their personal and professional identities They also enabled them to stay resilient in the face of hostile reactions from parents or colleagues, either in relation to their sexuality or in relation to the work they were doing in school to promote LGBTQ+ inclusion. In contrast to the others, Oliver's account demonstrates the tensions between religion and sexuality and highlights how these tensions can constrain teacher identity, agency, and restrict resilience.

Existing literature is dominated by accounts which position queer teachers as victims. We were concerned that this locates them within a victimized framework. Although the accounts that we have presented illuminate negative experiences, the narratives are largely positive, in contrast with existing literature. There is a need to re-conceptualize queer teachers to by locating their experiences within positive narratives which re-position them as resilient, skilled professionals who are active agents with potential to contribute to the advancement of inclusion and social justice within education. The repetition of victimized accounts which dominates existing literature only presents a partial account of the experiences of queer teachers. There is a need to create stories of empowerment which highlight the contribution that queer teachers can make to inclusion and social justice rather than repeating narratives of discrimination and prejudice.

## Data Availability Statement

The original contributions presented in the study are included in the article/supplementary material, further inquiries can be directed to the corresponding authors.

## Ethics Statement

The studies involving human participants were reviewed and approved by Leeds Trinity University Research Ethics Committee. The participants provided their written informed consent to participate in this study.

## Author Contributions

The authors worked together to prepare and format the study for this publication. Together, the authors approved and agreed the final draft.

## Conflict of Interest

The authors declare that the research was conducted in the absence of any commercial or financial relationships that could be construed as a potential conflict of interest.
